# The diverse roles of cysteine proteases in parasites and their suitability as drug targets

**DOI:** 10.1371/journal.pntd.0005639

**Published:** 2018-08-23

**Authors:** James H. McKerrow

**Affiliations:** Center for Discovery and Innovation in Parasitic Diseases, Skaggs School of Pharmacy and Pharmaceutical Sciences, University of California San Diego, La Jolla, California, United States of America; George Washington University School of Medicine and Health Sciences, UNITED STATES

Protozoan and metazoan parasites are the causative agents of many neglected diseases of global health import. Because these are diseases of poor people in resource-poor areas, there has been little economic incentive to develop new drugs in the pharmaceutical industry. Attention has therefore focused on suitable molecular targets in these parasites that could be exploited for new drugs at a relatively low cost. Cysteine proteases are particularly attractive for several reasons. They are ubiquitous in parasites; they are known to be druggable; and much is known about their structure, mechanism of action, and how to inhibit them. This compendium of reviews will focus attention on selected proteases and their function.

**Fig 1:**
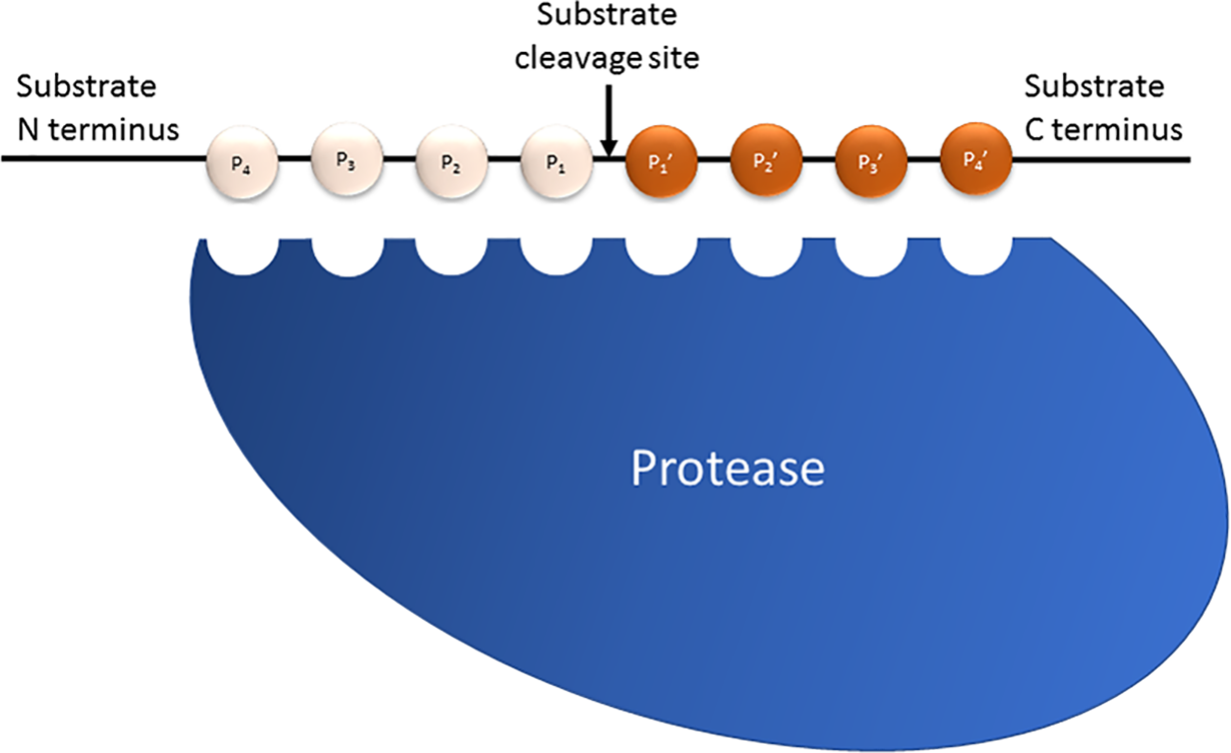
Protease substrate specificity in which P1 to P3 represent amino acid side chains from the site of peptide bond cleavage (hashed line) to N terminus of protein or peptide. P1’ to P3’ represent amino acid sidechains from cleavage site to C terminus. The nomenclature was first defined by Schecter and Berger [3].

Cysteine proteases are named because of the importance of a cysteine thiol group as the key nucleophile in the active site of the enzyme. The thiol group acts as a nucleophile in the initial steps of catalytic cleavage of the peptide bond (see [1,2] for a diagram). However, classification of cysteine proteases into distinct families is dependent both on sequence homology as well as on the specificity of the protease for amino acid sidechains in the “P1” position of the substrate (Fig 1). Remember, “cysteine” proteases are not named because they cleave a peptide bond next to a cysteine.

Several types of cysteine proteases are represented in the genomes of human and veterinary parasites. These include Clan CA (papain family) cysteine proteases, Clan CD asparaginyl endopeptidases (legumains), and caspases [1]. Whereas trypsin family serine proteases are the dominant gene family in vertebrates, Clan CA cysteine proteases are the dominant gene family of proteases in protozoa and metazoan [4,5]. This differential is particularly striking if one observes the proteases primarily involved in gut protein digestion. With the evolution of the pancreas, S1 serine proteases became the digestive enzymes, but invertebrates’ cysteine cathepsins still play that role. The accompanying reviews on helminth parasites drive home this point.

Because of the presence of an additional atomic “shell” in sulfur, the thiol group of cysteine is a much more effective nucleophile than the hydroxyl group of serine proteases. Therefore, primitive eukaryotes, like protozoa and metazoa, may have used a “more effective” family of proteases for their important biological and parasitic functions. However, this hypothesis also suggests a drawback. Cysteine proteases are more easily inactivated than serine proteases in an oxygen-rich environment. There was certainly a change in the relative abundance of serine versus cysteine protease genes in insects, mollusks, and crustaceans. The exact reasons for this shift remain unknown, but one might envision that the change reflected adaptation to terrestrial life with higher oxygen tension or the primacy of aerobic metabolism.

Clan CA (papain family) cysteine proteases are the focus of this review. This is not to suggest that other cysteine proteases of parasites are not suitable drug targets. In fact, much research is now centered on drug discovery targeting legumains, metacaspases, caspases, and the proteasome. As more information accrues, those proteases will also be reviewed. Likewise, the regulation of cysteine proteases in parasites is an important research focus, including work on protein inhibitors like chagasin [6].

Cysteine proteases are not unique to parasites [7,8]. Homologues can be found in vertebrates including animal and human hosts. However, there is an important and frequently overlooked difference. Human homologues, such as the cathepsins, are often found in high concentration in intracellular organelles like the lysosome. It has been estimated that in some cases, the concentration may be in the millimolar range [9]. In addition, there is redundancy in human cysteine proteases not found in the simpler parasite genomes. This is likely one reason why gene knockouts of cathepsins in mice are not lethal and, in fact, often have minimal consequences [10]. Therefore, in evaluating protease inhibitors as potential drugs, one must consider “biological selectivity” in addition to biochemical selectivity. In other words, a specific protease inhibitor may show no selectivity for parasite versus host protease targets but may still become an effective drug because of the location of the parasite target. Parasites are often in a more vulnerable location (like the bloodstream or intestinal lumen for helminth parasites). Or their proteases may be on their cell surface (as with *Trypanosoma cruzi* amastigotes). Furthermore, successful parasites must be able to scavenge small molecules from the host, so one might also hypothesize that protease inhibitors could be selectively taken up and concentrated in a parasite versus adjacent host tissue.
